# Content and Clinical Validation of the Nursing Outcome “Health Literacy Behaviour”: A Validation Protocol

**DOI:** 10.3390/healthcare11040481

**Published:** 2023-02-07

**Authors:** Alba Correro-Bermejo, Martina Fernández-Gutiérrez, Miriam Poza-Méndez, Pilar Bas-Sarmiento

**Affiliations:** 1 Punta Europa Hospital, Andalusian Health System, 11207 Algeciras, 11003 Cadiz, Spain; 2 Department of Nursing and Physiotherapy, University of Cadiz, 11009 Cadiz, Spain; 3 University Institute of Research in Social Sustainable Development (INDESS), University of Cadiz, 11406 Jerez de la Frontera, Spain; 4 Instituto de Investigación e Innovación Biomédica de Cádiz, INiBICA, 11009 Cadiz, Spain

**Keywords:** validation protocol, health literacy, Nursing Outcomes Classification (NOC), nursing, outcome assessment (health care)

## Abstract

Health literacy (HL) is a result of health promotion and education that has been included as a nursing intervention since 2013. It was proposed, as a nursing activity, to “determine health literacy status at initiation of contact with the patient through informal and/or formal assessments”. Because of that, the outcome ‘Health Literacy Behaviour’ has been incorporated in the sixth edition of the Nursing Outcomes Classification (NOC). It collects the patient’s different HL levels and allows them to be identified and evaluated in a social and health context. Nursing outcomes are helpful and provide relevant information for the evaluation of nursing interventions. Objectives: To validate the contents of the nursing outcome ‘Health Literacy Behaviour (2015)’ in order to use them in nursing care plans, and to evaluate their psychometric properties, application level, and effectiveness in nursing care to detect low health literacy patients. Methods: a methodological two-phased study: (1) an exploratory study and content validation by expert consensus, who will evaluate revised content of nursing outcomes; (2) methodological design by clinical validation. Conclusion: The validation of this nursing outcome in NOC will enable the generation of a helpful tool that would facilitate nurses to set individualised and efficient care interventions and identify low health literacy populations.

## 1. Introduction

Health literacy (HL) is a social determinant of health that depends on individual capacities and abilities, the cultural context, the health system, and is considered a step before patient empowerment [1,2,3,4,5].

In the European context, the Health Literacy European Project (HLS-EU-Project) has proposed a conceptual model for HL that includes both medical assistance and a public health perspective [6]. It also identifies the main factors affecting HL as well as the paths that link these factors to health outcomes. Health literacy has been defined as “the knowledge, motivation, and competencies to access, understand, appraise, and apply information, to make judgments and decisions in terms of healthcare, disease prevention, and healthy behaviors, to maintain and promote quality of life throughout the life course” [6].

This definition is based on a conceptual model that combines four dimensions referring to health information processing (the competencies related to the process of accessing, understanding, appraising, and applying health-related information) with the three levels of domains (healthcare, disease prevention, and health promotion) that yields a matrix with 12 dimensions of health literacy [6].

Low HL is associated with low participation in health promotion and disease detection activities, inadequate management of chronic diseases, poor understanding of complex treatments, errors in taking medication, increased hospitalisation and re-hospitalisation, increased misuse of emergency services, low use of preventive services, and an increase in morbimortality [6,7,8,9,10,11,12].

Today, more than ever, while we are facing an unprecedented pandemic, it is observed that the lack of knowledge in the population about health in general, and about COVID-19 in particular, is considered a public health problem worldwide. Curiously, most of the adult population confirms that they have health literacy problems and do not have sufficient skills to take care of their health and others’ [13]. The phenomenon, called ‘infodemic’, has emerged dangerously in the context of the COVID-19 pandemic, with disinformation appearing on the scene, including false or misleading information in digital and physical environments [13,14,15,16,17,18,19]. The excess of information, often contradictory, makes it difficult to find beneficial guidance for people and can hinder decision making in health. In this setting, health literacy becomes essential to distinguish quality information and to make informed health decisions [19,20,21].

One of the roles of nursing is to create a health-literate society that takes an active role in research, education, and health promotion. Strategies to educate the population should be incorporated into individual patient care plans to become part of nurses’ routine clinical practice [22,23]. The Nursing Outcome Classification (NOC) was born in 1991 at the University of Iowa, as a standardised grouping of patient results obtained in professional clinical practice that allows identifying, naming, classifying, and measuring the results and indicators that can be achieved through nursing interventions, based on their own and everyday language. This would lead us to have a scientific basis to modify behavioural guidelines regarding the care applied by nurses and facilitate the evaluation of the results to improve the quality of patient care. In short, the NOC has been designed to measure the effectiveness of nursing interventions. The NOC complements two other classifications, the North American Nursing Diagnosis Association International—NANDA-I, nursing diagnoses (ND), and the Nursing Interventions Classification—NIC, which groups nursing interventions and activities.

The fifth edition of the *Nursing Interventions Classification (NIC) Manual* incorporated the nursing intervention “Health Literacy Enhancement (5515)” defined as: “Assisting individuals with limited ability to obtain, process, and understand information related to health and illness”. It proposed, as a nursing activity, among others, to “determine health literacy status at initiation of contact with the patient through informal and/or formal assessments” [24]. Furthermore, in 2016, diagnosis 00262 “Readiness for enhanced health literacy” was published in *Nursing Diagnoses, Definitions, and Classification* of the North American Nursing Diagnosis Association (NANDA) as:

“A pattern of use and development of a set of skills and competencies (literacy, knowledge, motivation, culture, and language) aimed at finding, understanding, evaluating, and using health information and concepts, in order to make daily decisions linked with promoting and maintaining health, reduces risks related to health and increase, at a global level, the quality of life, which can be reinforced [25]”.

However, it was not until 2018 when a NOC outcome was proposed in this regard which allowed its evaluation [26]. NOC outcome ‘Health Literacy Behaviour’ is defined as “personal actions to obtain, understand, and evaluate information related to health, illness, and available services to make care decisions”. It is associated with 21 indicators that help identify the status in which a person is situated. The indicators of a NOC outcome are measured using five-point Likert-type scales, where the lowest score represents the most negative state of the patient and the highest score the best possible.

Its inclusion in the nursing taxonomy and care plans means that nurses can carry out more individualised and efficient care, allowing the evaluation and promotion of the patient’s autonomy to make decisions about their health, making them participate in activities that promote health and disease prevention, training them in self-care, and achieving positive therapeutic adherence. It is vital to execute individualised health education interventions, tools that facilitate systematic, objective, and reliable evaluation of the health literacy level of the population. Similarly, a need to determine the significance of the scores of the indicators of a NOC outcome has been detected, which demonstrates that this should be carried out in practice [27]. Therefore, the construction of operational definitions (OD), as well as their subsequent clinical validation, can assist in the process of validation of outcomes [28].

The evidence shows that there are two types of validation, content and clinical. Some studies focus solely on carrying out content validation [29], and others on carrying out the clinical [30]. However, there are authors who state [31,32,33,34] that, in order to obtain more reliable and accurate results, it is necessary to perform both types of validation.

There is no consensus among experts to establish a specific method for content validation of a test or instrument. Even so, many authors agree that in order to achieve a more accurate validation, it is necessary to have a sample of experts intentionally, knowing and analysing the characteristics of said sample and, on the other hand, to be clear about the dimensions that are to be evaluated in the instrument that can differ in each investigation [33,34,35]. Following these guidelines, we have followed Bellido et al.’s method [31,32] which uses the Delphi technique through two rounds of expert consensus and the Content Validity Index (CVI) which is obtained from the Lynn method [35]. Finally, to ensure the use of indicators and their definitions in clinical practice, it is necessary to verify the applicability of these in a real clinical environment.

The purpose of this project is to validate the adequacy of the content of NOC outcome ‘Health Literacy Behaviour’ for use in individualised nursing care plans, to evaluate its psychometric properties, and its applicability and efficacy in clinical nursing practice to detect patients with low HL.

## 2. Materials and Methods

A two-phased study: An exploratory study and content validation by expert consensus and methodological design by clinical validation.

### 2.1. Phase 1. Content Validation of NOC Outcome “Health Literacy Behaviour”

#### 2.1.1. Design

An exploratory and cross-sectional study method of content validity. The indicators of this NOC outcome have been previously defined operationally.

#### 2.1.2. Sample

A panel of at least 18 experts [31,36] will be selected using a convenience sampling among health literacy and nursing taxonomy experts from universities, nursing colleges, and/or nursing associations to assess the face and content validity of the indicators [37]. The following inclusion criteria will be established: (a) minimum clinical experience of at least two years; (b) minimum of 5 years of developing and using experience in the nursing taxonomy area; (c) to participate or have participated in research activities and have scientific academic productions in the areas related to taxonomies and/or instruments validation and/or Health Literacy; and (d) academic experience in the field of at least two years. To participate as an expert, you must meet criterion (c), as well as meet one more (a, b, or d). Refusal to participate in the study will be considered an exclusion [38,39]. Experts will be recruited by email through Professional Nursing Associations, Nursing Professional Colleges, and the universities of Spain. They will complete an anonymously survey through Google Forms.

#### 2.1.3. Variables

The following grouped variables will be considered.

Socio-demographic variables: sex, age, position in the institution where they work, seniority in the profession, level of study, academic degree, and whether they have a doctoral degree.

NOC outcome “Health Literacy Behaviour (2015)”: Table 1 shows the indicators included in the NOC outcome [26]. To evaluate each indicator, a 5-point Likert-type measurement scale will be used. Responses range from 1 (“no knowledge”) to 5 (“extensive knowledge”).

#### 2.1.4. Process

The indicators will be defined operationally based on the scientific literature. The content validity of the nursing outcome will be determined by submitting it to a panel of experts. Two consensus phases will be carried out using the Delphi technique [31,32]. In the first consensus round, the experts will be asked whether the indicators and their definitions (Table 1) are appropriate or not, using a four-point Likert-type scale (1 = Not appropriate, 2 = Somewhat appropriate, 3 = Fairly appropriate, and 4 = Totally appropriate). Simultaneously, completeness will also be measured, referring to whether the nursing outcome in question needs more indicators or whether it is nevertheless complete. Following Escobar and Cuervo [40], in the second round of consensus, each indicator and its definition will be measured: (a) Clarity: An indicator will be clear if it is “easily understood, that is, its syntactic and semantics are adequate”; (b) Relevance: An indicator will be relevant if “it is essential or important, that is, if it should be included”.

Both clarity and relevance will be measured with a 4-point Likert scale where 1 means “Not clear/irrelevant”; 2 = Unclear/ low relevant; 3 = Fairly clear/fairly relevant”; 4 = Totally clear/ totally relevant.

Coherence: if the indicator “has a logical relationship with any of the dimensions that are being measured”, the experts will classify which dimension of HL proposed by Sørensen et al. [6] belongs to each indicator.

### 2.2. Phase 2. Clinical Validation of NOC Outcome “Health Literacy Behaviour”

#### 2.2.1. Design

Methodological design of clinical validation.

#### 2.2.2. Sample

A sample of chronic patients from primary and specialised care will be considered. The sample size will be estimated by calculating five patients per indicator [29]. If the 21 initial indicators remained, 105 patients would be needed. Inclusion criteria will be: 18 years or older; attended by the Case Manager in a Primary Care Centre or Hospital of reference and/or family nurse and/or family doctor and/or specialist physician and/or Specialised Care nurse in Basic Health Zones; considered as chronic patients; and acceptance to participate in the study by signing a written informed consent. Exclusion criteria are patients who do not have sufficient cognitive and functional abilities to participate in the study or do not consent to participate.

#### 2.2.3. Variables

The following grouped variables will be considered:

Socio-demographic variables: sex, age, nationality, civil status, level of studies, and income.

Clinical variables: Main diagnosis; time of evolution after diagnosis; secondary chronic pathologies; the number of medical visits in the last three months (primary and specialised care); the number of visits to the primary care nurse in the last three months; the number of hospital admissions in the last three months; the number of visits to the case manager in the last three months.

NOC Outcome Health Literacy Behaviour Indicators: Operationalised (with their definitions) and non-operationalised.

Health Literacy: Health literacy level will be obtained by The European Health Literacy Survey Questionnaire (HLS-EU-Q16) [41]. The HLS-EU-Q16 contains 16 items addressing self-reported difficulties in accessing, understanding, appraising, and applying the information to tasks related to making decisions in health care, disease prevention, and health promotion. Each item is rated on a four-point Likert scale (very difficult, difficult, easy, and very easy) and a “do not know/no answer”. “Do not know or no answer” answers are coded as “no answer” [41]. Following the authors’ instructions, when scoring the HLS-EU-Q16, the categories “very difficult” and “difficult” are scored as 0, and the categories “easy” and “very easy” are scored as 1. Scale values are calculated as simply summed scores only for respondents who answered at least 14 items. Scoring varies between 0 and 16, establishing three levels of HL: inadequate (0–8), problematic (9–12), and sufficient (13–16) [41]. Currently, it is the only tool developed in our context supported by a well-established theoretical framework, with a solid conceptual base [6], taking into account the different dimensions of the HL [6,41,42,43,44].

#### 2.2.4. Process

Two nurses will be needed, one will pass the nursing outcome indicators proposed without the operational definitions, and the other will pass the questionnaire with the operational definitions [30].

#### 2.2.5. Data Analysis

Descriptive statistics will summarise the socio-demographic characteristics.

The data will be analysed using the Statistical Package for the Social Sciences (SPSS), version 22. A 5% significance level will be adopted for all tests.

The Content Validity Index (CVI) [33] method, following Bellido and Pancorbo’s experience [31], will be used to establish the content validity of the outcomes:

- Content Validity Index (CVI) of the indicators (CVI-i): the number of experts who give scores of 3 or 4 / number of experts who have issued scores.
- Content Validity Index (CVI) Universal Agreement Method (CVI-au): the number of indicators with CVI-i = 1 / total number of indicators.
- Content Validity Index (CVI) Average Method (CVI-p): average of the CVI-i of all indicators, establishing a value greater than 0 [33].

Factorial Validation Index (FVI): The degree to which experts identify each item with the dimension to which it belongs. The calculation is performed by indicator: Proportion of experts who correctly associate the item with its dimension, and global FVI: Average of the FVI of all items. Establishing the levels: No acceptable: FVI < 0.8; Acceptable: 0.80 ≤ FVI < 0.90; Excellent: FVI ≥ 0.90 [45].

To determine the psychometric properties of the NOC outcome developed in the second phase, the following will be analysed:

- Evaluation of reliability

Internal consistency: Cronbach’s alpha coefficient (α) and the item-total correlation (ITC) when an item is eliminated [46]—via Pearson’s correlation coefficient—will be used in order to evaluate the internal consistency. The scale will be considered to have adequate internal consistency if (1) the α scores range from 0.80 to 0.90 [47]; (2) the values of α when removing the item do not modify the reliability of the scale [48]; and (3) the ITC score, when an item is eliminated, is equal to or less than 0.40 [49].

Inter-observer Agreement: The intraclass correlation coefficient (ICC) of the NOC scores obtained in the NOC outcome in two administrations X days apart will be calculated. The maximum possible agreement would be reached when ICC = 1. In general, values below 0.4 indicate low reliability; when they are between 0.4 and 0.75, reliability between fair and good; and values greater than 0.75 refer to excellent reliability [50].

- Evaluation of validity

Factor Analysis: Exploratory factor analysis will be performed to uncover the underlying structure of the NOC indicators following the methodology proposed by Norman and Streiner [51]. The principal component analysis method will be used as a factor extraction procedure, and the Quartimax method will be selected as the rotation procedure to simplify the interpretation of the observed variables minimising the number of factors needed to explain the data. Previously, Kaiser–Meyer–Olkin measure and Bartlett’s sphericity test, the adequacy of the sample will be evaluated. The following criteria will be used to establish the appropriate number of factors and an appropriate factorial structure: (1) extraction of factors with their values (eigenvalues) greater than 1 [52]; (2) exploration of the Cattell scree plot searching for the point where there is a slope of the curve [53]; (3) each factor must contain at least three items since an inferior factor is not considered well-defined unless the highest weight of at least three variables is found in the factor [54]. A confirmatory factor analysis will be performed to verify the fitness of data to the factor structure found adequate in the previous analysis. The maximum likelihood estimation method and the covariance matrix between the items will be used for CFA. The fit indices proposed by Kline [55] will be considered to assess the fit of the data to the proposed model: (a) no statistical significance of the Chi-square test indicating that the model achieves a perfect fit with the observed data; (b) comparative fit index (CFI) ≥ 0.90 as indicative of a good fit [56]; (c) Tucker–Lewis Index (TLI ≥ 0.90 as suggestive of a good fit [57]; (d) root mean square error of approximation (RMSEA) ≤ 0.05 as indicative of an excellent fit and <0.08 of acceptable fit of the data [58]; (e) standardised root mean square residual (SRMR) ≤ 0.05 as suggestive of a good fit [59].

Known-groups validity: The overall NOC score will be compared between groups that will be presumed to differ in an attribute measured by the NOC outcome, HL, due to a known characteristic to determine the known-groups validity. In this case, these variables of characterisation will be “level of education” and “incomes” since these demographic attributes determine a differential response between education and HL level and between incomes and HL level [60,61]. An analysis of variance (ANOVA) or Kruskal–Wallis tests will be used, as appropriate, to compare the groups.

Convergent validity: To evaluate the degree to which the NOC outcome directly correlates with other instruments that measure the same construct, in this case, we will measure the correlation of our NOC outcome with the HLS-EU-Q16 questionnaire proposed by the HLS-EU Project. Pearson or Spearman test will be used, depending on whether the data will follow a normal distribution in the sample [62].

- Other non-metric properties assessment.

Applicability: The applicability of a scale refers to the ability of the scale to be used in real scenarios [63] and will be evaluated based on the time it takes to complete the scale. The estimated time to complete the questionnaire is 15 min.

## 3. Conclusions

The fifth edition of the *Nursing Interventions Classification (NIC) Manual* incorporated the nursing intervention “Health Literacy Enhancement (5515)”. It proposed, as a nursing activity, among others, to “determine health literacy status at initiation of contact with the patient through informal and/or formal assessments” [24]. However, to carry out these nursing interventions, it is necessary to have tools to measure the health literacy level of our patients and adapt the interventions to said level.

The validation of this NOC outcome will enable the generation of a helpful tool that would facilitate nurses to set individualised and efficient care interventions and identify low health literacy populations.

## 4. Ethics and Dissemination

This research will be carried out following the principles established in the current revised version of the Declaration of Helsinki [64] and following the applicable legal requirements for biomedical research [65] as a critical instrument for improving the quality and life expectancy of citizens and increasing their well-being.

Likewise, the signing of the informed consent will be requested to participate both the experts and the patients.

Outcomes from this study will include a health literacy tool that would facilitate nurses to set individualised and efficient care interventions and identify low health literacy populations. It may also have applicability across other chronic diseases. Using a multi-faceted strategy that targets local and national interdisciplinary audiences, findings, and outcomes will be disseminated across clinical and primary healthcare sectors and through policy decision-makers and patient organisations.

The findings will be disseminated to stakeholders by using various methods, including the lead author’s doctoral dissertation, peer-reviewed journals, academic conferences, and other verbal and digital communication channels.

## Figures and Tables

**Table 1:** NOC Outcome “Health Literacy Behaviour (2015)” indicators.

INDICATORS/OUTCOME OVERALL RATING	Never Demonstrated	Rarely Demonstrated	Sometimes Demonstrated	Often Demonstrated	Consistently Demonstrated	
(201501) Identifies personal health needs	1	2	3	4	5	N.A
(201502) Obtains reputable information relevant to health	1	2	3	4	5	N.A
(201503) Verbalises understanding of written information relevant to health	1	2	3	4	5	N.A
(201504) Verbalises understanding of verbal information relevant to health	1	2	3	4	5	N.A
(201505) Verbalises understanding of visual information relevant to health	1	2	3	4	5	N.A
(201506) Verbalises understanding of recommended medication	1	2	3	4	5	N.A
(201507) Verbalises understanding of recommended treatment	1	2	3	4	5	N.A
(201508) Evaluates information relevant to personal health	1	2	3	4	5	N.A
(201509) Acknowledges patient rights	1	2	3	4	5	N.A
(201510) Acknowledges patient responsibilities	1	2	3	4	5	N.A
(201511) Completes health-related documents	1	2	3	4	5	N.A
(201512) Identifies personal health care preferences	1	2	3	4	5	N.A
(201513) Identifies healthcare providers	1	2	3	4	5	N.A
(201514) Identifies preventive services	1	2	3	4	5	N.A
(201515) Shares questions	1	2	3	4	5	N.A
(201516) Shares concerns	1	2	3	4	5	N.A
(201517) Accesses healthcare services congruent with needs	1	2	3	4	5	N.A
(201518) Uses personal support system	1	2	3	4	5	N.A
(201519) Applies health information to their personal situation	1	2	3	4	5	N.A
(201520) Makes informed decisions about healthcare	1	2	3	4	5	N.A
(201521) Shares decisions regarding healthcare	1	2	3	4	5	N.A

Resource: NOC, Nursing Outcomes Classification, 2018.

## Data Availability

Not applicable.
